# Supplementary material for: Creatinine to Body Weight Ratio Is Associated with Incident Diabetes: Population-Based Cohort Study
Source: J Clin Med. 2020 Jan 15;9(1):227. doi: 10.3390/jcm9010227 (PMC7020036; doi:10.3390/jcm9010227)
Supplement: Supplementary file 1 [file jcm-09-00227-s001.pdf]

Journal of
Clinical Medicine

Supplemental Table 1. Clinical characteristics difference between the participants with and without follow-up.

**Men**	**Participants without follow-up n = 4113**	**Participants with follow-up n = 10,603**	***p***
Age (year)	45.5 (11.3)	45.8 (9.4)	0.064
Body weight (kg)	67.7 (10.4)	67.9 (10.1)	0.254
Height (cm)	170.5 (6.2)	170.5 (6.0)	0.749
Body mass index (kg/m^2)	23.2 (3.1)	23.3 (3.0)	0.134
Fasting plasma glucose (mmol/L)	5.4 (0.5)	5.4 (0.5)	0.966
Hemoglobin A1c (%)	5.3 (0.3)	5.2 (0.4)	<0.001
Hemoglobin A1c (mmol/L)	34.0 (3.7)	33.3 (3.9)	<0.001
Creatinine (mg/dL)	0.90 (0.12)	0.92 (0.12)	<0.001
Creatinine (umol/L)	79.7 (10.6)	81.7 (10.6)	<0.001
Triglycerides (mmol/L)	1.2 (0.9)	1.2 (0.9)	0.056
HDL cholesterol (mmol/L)	1.3 (0.4)	1.3 (0.3)	<0.001
Systolic blood pressure (mmHg)	121.4 (15.4)	121.0 (15.0)	0.190
Diastolic blood pressure (mmHg)	76.1 (10.7)	76.5 (10.3)	0.026
Exercise (-/+)	3,381/732	8,595/2,008	0.111
Smoking (Non/Past/Current)	1,245/1,242/1,626	3,295/3,455/3,853	<0.001
Alcohol intake (Non-min/light/moderate/heavy)	2,510/572/514/517	6,175/1,644/1,495/1,289	0.002
Cre/BW ratio	0.014 (0.003)	0.014 (0.002)	<0.001
**Women**	**Non n = 3348**	**Participants n = 7586**	***p***
Age (year)	44.5 (11.2)	44.3 (9.2)	0.380
Body weight (kg)	52.6 (8.0)	53.0 (8.2)	0.015
Height (cm)	157.8 (5.6)	158.1 (5.4)	0.017
Body mass index (kg/m^2)	21.1 (3.0)	21.2 (3.1)	0.169
Fasting plasma glucose (mmol/L)	5.1 (0.4)	5.0 (0.4)	<0.001
Hemoglobin A1c (%)	5.2 (0.3)	5.2 (0.3)	<0.001
Hemoglobin A1c (mmol/L)	33.8 (3.6)	33.3 (3.8)	<0.001
Creatinine (mg/dL)	0.66 (0.10)	0.68 (0.10)	<0.001
Creatinine (umol/L)	58.7 (8.8)	59.9 (9.1)	<0.001
Triglycerides (mmol/L)	0.7 (0.5)	0.7 (0.5)	0.578
HDL cholesterol (mmol/L)	1.7 (0.4)	1.6 (0.4)	<0.001
Systolic blood pressure (mmHg)	111.8 (15.7)	110.9 (15.4)	0.011
Diastolic blood pressure (mmHg)	68.8 (10.5)	68.7 (10.3)	0.854
Exercise (-/+)	2,806//542	6,323/1,263	0.550
Smoking (Non/Past/Current)	2,772/277/299	6,543/514/529	<0.001
Alcohol intake (Non-min/light/moderate/heavy)	3,044/151/100/53	6,857/425/215/89	0.036
Cre/BW ratio	0.013 (0.002)	0.013 (0.003)	0.001

Cre/BW, Creatinine to body weight; HDL, high density lipoprotein. Continues variables are expressed as mean (SD), and the differences are evaluated by student's t test. Categorize variables are expressed as number, and the differences are evaluated by Chi-squared test.
